# The Territorial Regimental Surgeon and Sanitary Instruction

**Published:** 1909-04-03

**Authors:** 


### THE ROYAL ARMY MEDICAL CORPS SECTION.

### THE TERRITORIAL REGIMENTAL SURGEON AND SANITARY INSTRUCTION.

In addition to training the regimental stretcher-bearers in a knowledge of first-aid, it is incumbent on the Territorial regimental surgeon to give instruction in military sanitation and prevention of disease to the sanitary sections, and possibly also to the water duty men of his unit. Further, it is expected of him that he should by means of demonstrations, or in any other way that seems to him advisable, strive to awaken in the combatant officers, and in all other ranks of the regiment an interest in, and knowledge of military hygiene, so as to enlist their willing co-operation in his efforts to maintain their health and consequent efficiency. In each of these directions the regimental surgeon may do good work.

As already mentioned in a previous article, the sanitary section of a regiment consists of a sergeant and eight men. They may be taken from the regimental pioneers, or they may be furnished by the various companies of the unit. From whichever source obtained care should be taken in their selection, as their duties are of a special kind, and men of good common sense and some strength of character are needed. In carrying out their training the scope and nature of the instruction should be adapted to their standard of intelligence and education, and be calculated to arouse in them interest in their work. To be successful it should be a combination of lectures and practical demonstrations.

The course of lectures given should, following the Manual of Sanitation, include the following special subjects:—

1. The importance of sanitation to an army.
2. The causes of disease.
3. The diseases of the soldier in the field.
4. The prevention of disease.
5. The sanitation of camps.
6. Sanitation on active service, on field days, and during manœuvres.

Under all these headings there is ample material for interesting and instructive lectures. Our campaigns in the past furnish plenty of object lessons of the enormous losses that may occur from what are really preventable diseases by disregard of the laws of sanitation. On the other hand it can be made quite clear and certain that modern advances in the science of sanitation and our wider knowledge of the causes of disease enable us to deal successfully with the special dangers to which an army in the field is exposed. Nowhere is that better demonstrated than in connection with enteric fever and dysentery, which have always been the most fatal diseases in past campaigns. We now know that they can be entirely kept in control by proper and perfect disposal of excreta, and by ensuring a supply of pure food and water. It is by bringing home these facts to the members of the sanitary sections that they will be brought to realise the important part they play in war.

It is, however, necessary that instruction such as that outlined above should be supplemented by some practical work. Sanitary sections should have opportunities given them of making instructional latrines, of building refuse destructors, and of constructing the different kinds of grease pits. Facilities for such practical work might be furnished by having a march out of the regiment into the country for the purpose of planning a regimental camp. This would allow of the selection and preparation, under the regimental surgeon and quartermaster, of sites for kitchens, ablution places, and latrines, while refuse destructors might be built and grease pits constructed. The importance, too, of a pure water supply might be demonstrated by placing one or more men over it, and thus controlling its use. After carrying through the above work everything should be replaced and put in order, thus furnishing the very necessary object-lesson that every camp should be properly cleaned before troops march out of it. The experience gained by such an afternoon's work would be invaluable, not only to the sanitary sections but to all ranks of the battalion, and regimental surgeons should urge it on the commanding officers of their units. Should, however, such a field day as suggested not be obtainable, another plan would be to arrange for a practical demonstration of the above details through the divisional school of instruction, by making application to the administrative medical officer of the division. In connection with the training of these sanitary sections, it should be remembered by the regimental surgeon that if he happens to be in a large town where his battalion forms part of a brigade, the medical officers of the regiments in that brigade may combine to hold one special class at which all the sanitary sections will attend. Under this arrangement they will carry on the class conjointly, each one taking up a special subject. They are also allowed to arrange for lectures by outside specialists, if thought advisable.

Although the present idea is that the training of the water-duty men should be carried out by the divisional school of instruction, this may not be the case, and it may fall upon the regimental surgeon. The conduct of such a class should be in accordance with the official memorandum that has been issued for the guidance of officers and others instructing men of the Territorial force in water section duties. The memorandum is so full and detailed that no comments on it are necessary. The five lectures that it embodies, embrace full instruction on water-borne diseases, on the sources and prevention of water pollution, on the purification of impure water and on the practical working of the new filter cart, a full description of which was given in The Hospital, for November 28th, 1908. As has been already insisted on in previous articles, no matter what the class to be instructed, successful results can only be obtained from it if the interest of the regimental combatant officers can be obtained in its working. If they can be got to understand that water sterilisation, the disposal of excreta, the personal hygiene of the men, and everything connected with the sanitation of the unit are not the sole work of the regimental surgeon, but should be equally shared by them, then the object aimed at will be attained. Let all ranks realise that they have not only to combat the human enemy opposed to them, but also the more deadly, because unseen microbes of disease, and they will listen to the instruction given them, and by their hearty co-operation ensure freedom from disease and increased fighting efficiency. In his work of tuition the regimental surgeon will obtain valuable assistance from the official "Manual of Sanitation," from Elkington's "Notes on Military Sanitation," from Caldwell's "Prevention of Disease in Armies in the Field," and "Military Hygiene," by R. H. Firth.

